# 3D-Printed Microfluidic Perfusion System for Parallel Monitoring of Hydrogel-Embedded Cell Cultures

**DOI:** 10.3390/cells12141816

**Published:** 2023-07-09

**Authors:** Katharina V. Meyer, Steffen Winkler, Pascal Lienig, Gerald Dräger, Janina Bahnemann

**Affiliations:** 1 Institute of Technical Chemistry, Leibniz University Hannover, 30167 Hannover, Germany; meyer@iftc.uni-hannover.de; 2 Institute of Physics, University of Augsburg, 86159 Augsburg, Germany; steffen.winkler@uni-a.de; 3 Institute of Organic Chemistry, Leibniz University Hannover, 30167 Hannover, Germany; pascal.lienig@oci.uni-hannover.de (P.L.); gerald.draeger@oci.uni-hannover.de (G.D.); 4 Centre for Advanced Analytics and Predictive Sciences (CAAPS), University of Augsburg, 86159 Augsburg, Germany

**Keywords:** 3D cell culture, organ-on-chip, membrane integration, 3D printing, hydrogel, microfluidic perfusion system

## Abstract

The use of three-dimensional (3D) cell cultures has become increasingly popular in the contexts of drug discovery, disease modelling, and tissue engineering, as they aim to replicate in vivo-like conditions. To achieve this, new hydrogels are being developed to mimic the extracellular matrix. Testing the ability of these hydrogels is crucial, and the presented 3D-printed microfluidic perfusion system offers a novel solution for the parallel cultivation and evaluation of four separate 3D cell cultures. This system enables easy microscopic monitoring of the hydrogel-embedded cells and significantly reduces the required volumes of hydrogel and cell suspension. This cultivation device is comprised of two 3D-printed parts, which provide four cell-containing hydrogel chambers and the associated perfusion medium chambers. An interfacing porous membrane ensures a defined hydrogel thickness and prevents flow-induced hydrogel detachment. Integrated microfluidic channels connect the perfusion chambers to the overall perfusion system, which can be operated in a standard CO_2_-incubator. A 3D-printed adapter ensures the compatibility of the cultivation device with standard imaging systems. Cultivation and cell staining experiments with hydrogel-embedded murine fibroblasts confirmed that cell morphology, viability, and growth inside this cultivation device are comparable with those observed within standard 96-well plates. Due to the high degree of customization offered by additive manufacturing, this system has great potential to be used as a customizable platform for 3D cell culture applications.

## 1. Introduction

The cultivation of cells in a laboratory is an essential and fundamental tool used across a wide array of scientific research fields. Traditionally, adherent cells have been cultivated as a monolayer, since protocols for the proper handling and analysis of such cultures are well-established [1]. However, in recent years it has become increasingly apparent that traditional monolayer cultures simply cannot be induced to mimic the three-dimensional (3D) environment that cells are exposed to in vivo—resulting in critical differences between 2D and 3D cell cultures in terms of their morphology, differentiation, protein expression, functionality, migration, apoptosis, and response to drugs [1,2,3,4,5,6]. As a result, 3D cell cultures are increasingly being favored by researchers due to their superior physiological relevance [7].

Today, 3D cell cultures are often achieved by using various scaffold-free techniques—for example, via low-adhesion plates which promote the aggregation of cells into spheroids, or via extracellular matrix (ECM) coated plates that facilitate cell differentiation into organoids [7]. The multilayers of cells obtained through these various mechanisms reflect the in vivo conditions more accurately than traditional 2D cultures, however, they are comparatively much more variable in terms of their size, shape, and composition. Furthermore, tracking and analyzing specific cells over an extended period of time becomes a far more difficult task, due to their potentially shifting position within this complex cell construct [7,8].

In vivo, however, most mammalian cells proliferate while surrounded by an ECM which forms a complex scaffold consisting primarily of hydrated proteins [1]. Taking a cue from nature, scaffold-based 3D culture approaches use fabricated 3D structures to imitate this ECM. Rigid scaffolds (for example, those fabricated from paper or fibrils) need to be laboriously synthesized and manufactured—and even once they are manufactured, seeding cells evenly and homogeneously within these structures often presents a substantial challenge for researchers. These issues make it difficult to truly standardize results between different experiments [7]. Furthermore, such rigid scaffolds are only suitable for certain types of tissue; they are patently unsuitable for use in myocardial tissue engineering, for example [9].

Hydrogels are able to mimic this natural ECM system by absorbing high amounts of water, and they also have the benefit of providing viscoelastic strength while facilitating a far more uniform distribution of cells [7,9]. Perhaps not surprisingly, there are now many materials available to researchers looking to “fine tune” the porosity, stiffness, and/or degradation of a hydrogel in order to optimize cell proliferation and better mimic organ-specific ECMs [6,10,11]. However, cell structure, function, and behavior are not merely influenced by the 3D arrangement of the cells—they are also influenced by the complex combination of biochemical, physical, and physicochemical properties (e.g., soluble factors, pH, oxygen supply, temperature and osmolality) which form the microenvironment in which the cell develops [12]. Exercising granular control over this cell microenvironment is thus of critical importance in many cases—particularly as it has been shown that (for example) stem cell development is influenced by this microenvironment and abnormal levels of pH and oxygen tension are associated with the development of various pathologies [12]. Furthermore, gradients in the cell microenvironment can act as signals influencing the regulation of cell function and behavior [12,13].

Microfluidic cell culture devices enable good control over the microenvironment of the cells and even the implementation of stable gradients of various forms in physiologically relevant scales [12]. Furthermore, microfluidic devices enable tissue-tissue communication, dynamic fluid flow, and the application of normal mechanical stimuli/cues [14]. 3D cell cultures within microfluidic devices can therefore even be used to mimic organs in their complex microarchitecture and function. These organ-on-chip (OOC) devices can, for example, be applied to improve the understanding of human drug metabolism and toxicity in vitro prior to the initiation of clinical trials [14,15,16,17]. Such OOC devices have, inter alia, already been successfully implemented for human livers [15], lungs [18], and even for modeling specific diseases such as virus-induced kidney disease [19] and an infected epidermis model, among others [20].

There is a downside, however: the fabrication of microfluidic devices using conventional methods can be very challenging, time consuming and expensive. One way to overcome these challenges is 3D printing, which is becoming increasingly popular within the field of biotechnology [21,22,23,24,25]. 3D printing not only offers researchers the ability to engage in rapid prototyping, but also permits the fabrication of highly customized complex structures [21]. It enables easy chip-to-world interfacing (such as 3D-printed Luer-lock-systems) for medium supply, as well as the use of removable support materials to facilitate the fabrication of overhanging structures and cavities (such as hollow microfluidic channels) [21,26].

As researchers continue to push for ever-more-realistic in vivo-like conditions within the laboratory, growing ambitions to construct more complex microenvironments for cell cultures will undoubtedly continue to spur on the development of novel hydrogels and the integration of various cell types. This will in turn create an ever-growing demand for screening systems that allow for more reliable evaluations of different cultivation conditions—including hydrogel compositions, cell densities, media supplements, oxygen concentrations, and other crucial factors which influence the cellular microenvironment. The 3D-printed microfluidic perfusion system presented in this study introduces a novel solution for the integration of hydrogels and parallel cultivation of four separate 3D cell cultures. By using a porous membrane for separation of the hydrogel compartment from the microfluidic perfusion channel system, the hydrogel of each chamber is constructed to be of an equal thickness and is protected from flow-induced detachment. In addition, this design also provides for comparatively easy monitoring of the cells. Finally, due to the high degree of customizability offered by additive manufacturing, this microfluidic cultivation system can be rapidly scaled to a multi-chamber device or a parallel perfusion system in a single device.

## 2. Materials and Methods

### 2.1. 3D Printing and Post-Processing of Cultivation Device and Perfusion System Parts

After computer-aided design (CAD) using SolidWorks 2022 (Dassault Systems Deutschland GmbH, Stuttgart, Germany), the 3D-printed parts of the cultivation device and perfusion system were fabricated using a high-resolution 3D printer AGILISTA-3200 W (Keyence Deutschland GmbH, Neu-Isenburg, Germany) which manufactures objects via inkjet technology using an ultraviolet (UV) curing process—resulting in a resolution of 635 × 400 dots per inch and a layer thickness of 15 µm [27]. The clear polyacrylate 3D printing material AR-M2 (Keyence Deutschland GmbH, Neu-Isenburg, Germany) was used as model material, and AR-S1 (Keyence Deutschland GmbH, Neu-Isenburg, Germany) was used as support material during the printing process. In its cured form, the model material shows biocompatibility in accordance with ISO 10993:12 [28]. Subsequent to the printing process, objects were scraped from the printing platform and the support material was removed via incubation in an ultrasonic water bath (Bandelin electronic, Berlin, Germany) for 30 min at 60 °C—twice with detergent (Fairy Ultra Plus, Procter and Gamble, Bethel, CT, USA), and then once more with ddH_2_O (Arium^®^ Sartorius Stedim Biotech GmbH, Göttingen, Germany)). Small channels were thoroughly rinsed after every incubation step by attaching a cleaning syringe. Finally, the objects were incubated in ethanol (70% *v*/*v*; VWR International GmbH, Darmstadt, Germany) on a SSM3 gyratory rocker (Cole-Parmer Instrument Company Ltd., St Neots, UK) at 70 rpm for 1 h, thoroughly rinsed with ddH_2_O, and then completely dried.

### 2.2. Assembly of the Cell Cultivation Device

The cultivation device consists of two 3D-printed parts separated by polyester membranes cut from Transwell^®^-Clear Inserts (pore size: 3 µm; Corning, Kaiserslautern, Germany), which are sealed via standard O-rings (Landefeld Druckluft und Hydraulik GmbH, Kassel, Germany; 6 × 1 mm, FKM). A transparent 0.25 mm thin polycarbonate sheet (Modulor GmbH, Berlin, Germany) was used as the bottom plate of the device. The polycarbonate sheet and the lower 3D-printed part of the system were bonded using an adhesive medical tape (3M 9877, 3M Medical Solutions Division, Healthcare Business Group, Neuss, Germany) and connected to the upper 3D-printed part using standard M2 metal screws and a custom-built metal frame. The adhesive medical tape was cropped using a cutting plotter (Cameo 4, Silhouette America, Inc., Lindon, UT, USA).

### 2.3. Assembly of the Perfusion System

Perfusion of the cell cultivation device was achieved from a medium reservoir with an IP-4 peristaltic pump (Ismatec, Wertheim, Germany) using Tygon^®^ pump tubing (IDEX Health and Science GmbH, Wertheim, Germany; inner Ø 1.22 mm), connecting standard chromatography PTFE tubing (Ø 0.8 mm, Bohlender GmbH, Grünsfeld, Germany) and fittings. A TubeSpin^®^ Bioreactor (TPP Techno Plastic Products AG, Trasadingen, Switzerland) was used as a medium reservoir. A 3D-printed adapter was designed to connect the tubing to the TubeSpin^®^ Bioreactor. For some experiments, a bubble trap with an internal volume of 97 µL (Darwin Microfluidics, Paris, France) was integrated into the perfusion setup and then used in passive mode.

### 2.4. Cell Line and Cell Culture Conditions

L-929 cells (CLS Cell Lines Services GmbH, Eppelheim, Germany) were routinely cultivated in Dulbecco’s Modified Eagle’s Medium (DMEM; Sigma-Aldrich Chemie GmbH, Steinheim, Germany), supplemented with 10% fetal calf serum (Sigma-Aldrich Chemie GmbH, Steinheim, Germany) and 1% Penicillin/Streptomycin (Sigma-Aldrich Chemie GmbH, Steinheim, Germany) within 175 cm^2^ cell culture flasks (Corning, CellBind Surface, Corning, NY, USA) in a 5% CO_2_ humidified atmosphere at 37 °C (Heracell 240 incubator, Thermo Fisher Scientific Inc., Waltham, MA, USA). At 70–85%, confluency, cells were harvested via Trypsin/EDTA solution treatment (Biochrom GmbH, Berlin, Germany). Experiments were performed with cells of passage numbers up to 10.

### 2.5. Hydrogel Preperation

For all experiments, an in situ cross-linkable alginate hydrogel described by Dahlmann et al. was used [9]. Briefly, the alginate hydrogel formation is based on the spontaneous condensation of an alginate hydrazide and an alginate aldehyde. Alginate aldehydes were generated via oxidation of vicinal diols within the monomeric units according to the Malaprade reaction. The carboxylate functions of alginate were directly transferred into the corresponding acyl hydrazides using standard carbodiimide chemistry [9]. Aldehydes were thoroughly dialyzed over a period of 3–5 days against distilled water, with a minimum repeated water exchange of three times per day. Hydrazides were dialyzed against 100 g·L^−1^ sodium chloride (NaCl; VWR International BVBA, Leuven, Belgium) for one day, followed by 50 g·L^−1^ NaCl for one day and two days against distilled water. Lyophilization was performed with a Christ Alpha 2–4 (Christ Osterode, Osterode am Harz, Germany) freeze dryer [9]. To begin a cultivation, both lyophilized hydrogel precursors were each dissolved in a 0.9% (*w*/*v*) NaCl solution at 70 °C to a concentration of 1% (*w*/*v*) using a Thermomixer (Thermomixer comfort, Eppendorf, Hamburg, Deutschland) at 1000 rpm, and then sterile filtered (0.2 µm PES syringe filter, Filtropur S, Sarstedt AG & Co. KG, Nümbrecht, Germany). Subsequently, volume fractions of 40% collagen I solution from bovine skin (3 mg·mL^−1^ aqueous solution in 0.01 M HCl, Sigma-Aldrich, Co., St. Louis, MO, USA), 5% 0.9% (*w*/*v*) NaCl solution and 5% 0.1 M sodium hydroxide (NaOH; Sigma-Aldrich Chemie GmbH, Steinheim, Germany) were mixed thoroughly with 25% sterile hydrazide-derivatives of alginate, and then kept on ice. Polymerization was initiated by adding 25% sterile alginate aldehyde and thereby obtaining a final gel concentration of 0.5% (*w*/*v*). Samples were thoroughly mixed and immediately transferred to the respective chamber or well. For cell-containing hydrogels, the required volume of cell suspension was centrifuged at 300× *g* for 5 min (MiniSpin^®^ plus, Eppendorf SE, Hamburg, Germany), the supernatant was discarded, and the cells were then resuspended in the sterile alginate aldehyde solution to obtain a final cell concentration of 0.75 million cells per mL.

### 2.6. Microscopic Analysis and Live/Dead Staining

Microscopic imaging was performed using a Cytation 5 Cell Imaging Multi-Mode Reader (BioTek Instruments GmbH, Bad Friedrichshall, Germany) at 37 °C. Compatibility of the cell cultivation device with standard imaging systems was ensured using a 3D-printed adapter in well plate format, which was designed using SolidWorks 2022 and fabricated from polylactic acid (PLA) filament (1.75 mm, Das Filament Inh. Roman Stieben, Emskirchen, Germany) with a Prusa i3 MK3 (Prusa Research a.s., Prague, Czech Republic). For imaging of the cultivation device, it was first removed from the perfusion system in a sterile environment, closed with 3D-printed blind plugs, and finally placed on its adapter in well plate format and transferred to the imaging system. For brightfield imaging, the intrinsic auto-exposure function of the Gen5 imaging software (Version 3.10.06, BioTek Instruments GmbH, Bad Friedrichshall, Germany) was used with 4× and 20× objectives. Live/dead staining of the cultivated cells was performed with calcein AM (Merck Chemicals GmbH, DE, USA) and propidium iodide (PI; Merck KGaA, Darmstadt, Germany). Cells inside the cultivation device were stained for 2 h, and cells cultivated in well plates were stained for 20 min with calcein AM (1 μM) and PI (1 μg·mL^−1^) containing phosphate-buffered saline (PBS; Life Technologies Limited, Paisley, UK) solution at 37 °C in the dark. Subsequently, the staining solution was removed, and samples were covered with dye-free PBS solution. For image analysis, stitching, and channel overlay pictures, the intrinsic functions of the Gen5 imaging software were used.

### 2.7. Perfusion Experiments

Assembly of the cultivation device at the beginning of a perfusion experiment and handling of the adapter in well plate format for imaging of the hydrogel-embedded cells is shown in the Video S5 in the supplementary material. Prior to an experiment, all tubing, fittings, and the metal screws/metal frame/sealings of the device were sterilized via autoclaving (30 min, 121 °C; Systec VX-150, Systec GmbH, Linden, Germany). The 3D-printed parts of the cultivation device as well as all parts of the bubble trap were disinfected via incubation in ethanol (70% *v*/*v*; VWR International GmbH, Darmstadt, Germany) on a SSM3 gyratory rocker (Cole-Parmer Instrument Company Ltd., St Neots, UK) at 70 rpm for 1 h, thoroughly rinsed with sterile ddH_2_O (Arium^®^ Sartorius Stedim Biotech GmbH, Göttingen, Germany), and completely dried in a sterile environment. For some experiments, the whole perfusion system filled with 25 mL cell culture medium and the assembled cell cultivation device, without membranes, were placed in the incubator (Heracell^TM^ VIOS 160i CO_2_ incubator, Thermo Fisher Scientific Inc., Waltham, MA, USA) overnight prior to beginning the experiment, in order to avoid the formation of gas bubbles inside the cultivation device during the cultivation process. To begin an experiment, the prepared cell cultivation device was disassembled in a sterile environment and every cultivation chamber was filled separately with hydrogel as described in Section 2.5. Subsequently, the membranes were placed on the hydrogel and the cultivation device was reassembled. The perfusion medium chambers were filled with the cultivation medium via a syringe before microscopic imaging of the hydrogel embedded cells was performed as described in Section 2.6. Afterwards, the cultivation device was connected to the perfusion system, placed in the incubator, and perfused with 0.25 mL·min^−1^ cultivation medium for three days. As a control experiment, 50 µL of the same cell containing hydrogel were cultivated in 96-well plates (Sarstedt AG and Co. KG, Nümbrecht, Germany) with 150 µL cultivation medium, and cells from the same cell suspension were seeded in the 96-well plate at a density of 7500 cells per well in 200 μL cell culture medium. All control experiments were conducted in at least triplicate.

## 3. Results

### 3.1. Design of the Microfluidic Cell Cultivation Device and Perfusion System

**Figure 1:**
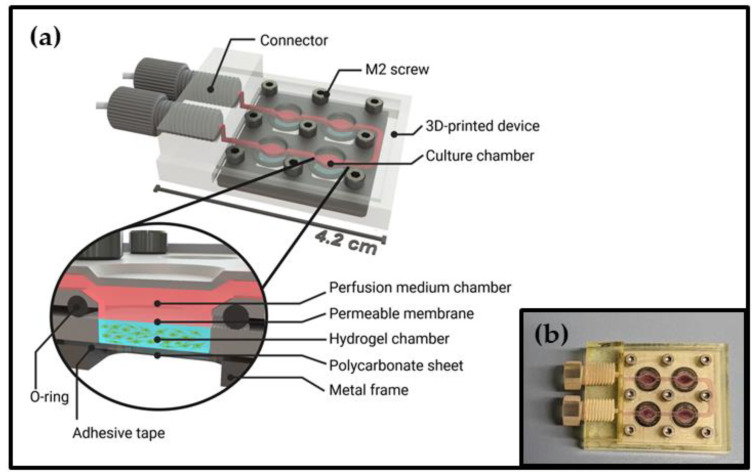
(**a**) Schematic illustration of the microfluidic cultivation device. The perfusion medium chambers and the cell-containing hydrogel chambers are separated by permeable membranes preventing detachment of the hydrogel. A transparent bottom sheet enables microscopic analysis of the hydrogel-embedded cells. (3D design was performed using SolidWorks 2022 CAD software; figure created with BioRender.com, accessed on 5 June 2023); (**b**) Picture of the assembled cultivation device filled with culture medium and closed with 3D-printed blind plugs.

The design of the microfluidic cell cultivation device is presented in Figure 1. The device was formed by two 3D-printed parts which created four cell-containing hydrogel chambers and their associated perfusion medium chambers—with volumes of 11.7 µL and 10.6 µL, respectively. The oval shape of the chambers ensured sufficient light incidence for microscopic examination of the cells, while also simultaneously enabling an adequate exchange of the perfusion medium. The perfusion medium chambers were connected to each other, as well as to the inlet and outlet of the device, via 0.7 × 1.0 mm channels. A more detailed illustration of the device design can be found in technical drawings in the supplementary material (Figures S1 and S2). Hydrogel chambers and perfusion medium chambers are separated by permeable membranes with a pore size of 3 µm, which ensure adequate gas exchange and sufficient nutrient supply to the cells while still separating the hydrogel from the microfluidic perfusion channel system. The use of a transparent 0.25 mm thin polycarbonate sheet to seal the bottom of the hydrogel chambers permitted microscopic examination of the hydrogel-embedded cells. The polycarbonate sheet and the lower 3D-printed part of the system were bonded using an adhesive medical tape, while the upper 3D-printed part was connected using nine standard M2 metal screws and a metal frame. This ensured easy assembly of the device and also enabled the extraction of the hydrogel and cells following the completion of an experiment. Since layer-by-layer manufacturing of the 3D-printed parts results in a high surface roughness, standard O-rings are used to ensure leak-proof sealing of the chambers. The spatial arrangement of the nine screws that were utilized ensured an equal distribution of the contact pressure. Using female threads as connection ports for standard chromatography fittings at the device-to-world-interface also enabled easy sealing with blind plugs for microscopy as well as easy connection of the device to the perfusion system.

**Figure 2:**
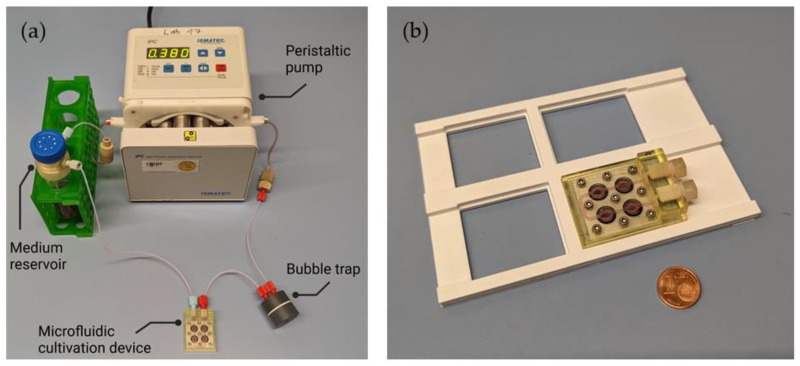
(**a**) Setup of the 3D-printed microfluidic perfusion system with cultivation device and bubble trap. The use of standard chromatography fittings as the device-to-world-interface enables easy connection of the device to the perfusion system. A bubble trap prevents gas bubbles inside the culture chambers; (**b**) Picture of the assembled cultivation device filled with medium and placed on its 3D-printed adapter in well plate format.

The assembled perfusion system—including the microfluidic cell cultivation device—is shown in Figure 2. The system consists of a medium reservoir, a peristaltic pump, tubing, and fittings, and can be operated in a standard CO_2_-incubator. A 3D-printed adapter was placed between the medium reservoir and its lid for sterile connection of the reservoir to the perfusion system tubing. A detailed illustration of the adapter can be found in a technical drawing in the supplementary material (Figure S3). A filter in the lid of the medium reservoir allowed for gas exchange to occur between the medium and the incubator interior. The cultivation medium was pumped cyclically from the reservoir into the cultivation device, and then back into the reservoir, at a speed of 250 µL·min^−1^. Integrating a bubble trap into the perfusion setup right before the cultivation device avoided the introduction of any air bubbles in the perfusion medium chambers (which could have potentially reduced comparability between the chambers and impaired the supply of nutrients and gases to the cells). Compatibility of the cell cultivation device with standard imaging systems was ensured via a 3D-printed adapter in well plate format, thereby ensuring exact positioning and a defined focal height (see Figure 2b).

### 3.2. Cultivation and Imaging of Hydrogel-Embedded Cells

L929 cells are commonly used for in vitro biocompatibility and cytotoxicity screenings, and they were therefore chosen as a suitable cell line for use in a proof-of-concept cultivation within this 3D-printed microfluidic cell cultivation device [28,29]. These cells were embedded in the hydrogel and cultivated over the course of three days in standard 96-well plates, as well as perfused into the chambers of the cultivation device. The experiment described herein was performed three times. In situ cross-linking of the hydrogel in the presence of L929 cells was sufficiently fast to avoid settling of the cells. At the beginning of the cultivation, an even distribution of the cells inside the hydrogel was microscopically verified in each microchamber, as well as the correct positioning of the membranes themselves. Throughout the cultivation process, no loss of the hydrogel was observed, indicating that potential hydrogel detachment induced by medium flow and/or cell-induced deformation of the hydrogel was prevented by the membranes. Furthermore, proper acclimatization of the whole perfusion system (including cell culture medium) in the incubator overnight prior to an experiment, as well as the integration of a bubble trap, avoided the formation of gas bubbles inside the cultivation device. This ensured that a consistent gas exchange and adequate supply of nutrients was provided to cells in every part of the cultivation chamber throughout the process.

**Figure 3:**
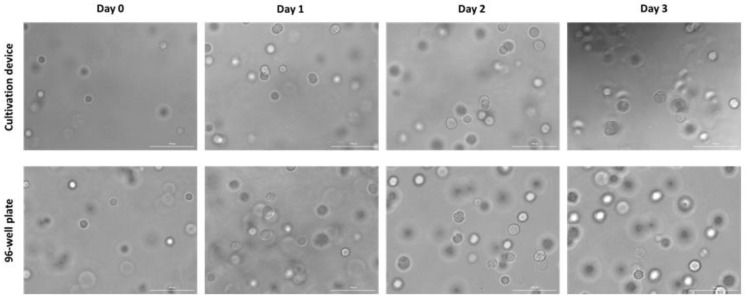
Representative brightfield images of hydrogel-embedded L929 cells in a cultivation chamber of the 3D-printed cell cultivation device and a well of a 96-well plate over the course of a cultivation. A similar morphology and growth of the cells could be observed in both cultivation vessels. Scale bar corresponds to 100 µm.

The design of the cultivation device and its adapter in well plate format allowed for imaging of every position inside the cultivation chambers (see z-stack video and montage image in the Supplementary Material [Video S6, Figure S4]). Figure 3 shows representative microscopic bright field images of the cells on each day of cultivation. The cells cultured in the chambers of the cultivation device and in the well plate displayed a similar morphology and growth over time. Cell spreading was not observed for the present hydrogel—resulting in round cell morphology in both cultivation vessels. However, cell division and slight deformation of the initial round cell shape was observed, indicative of a viable and growing culture. The growth behavior of the individual cells did not appear to be dependent upon their position within the cultivation chamber (Video S6, Figure S4).

**Figure 4:**
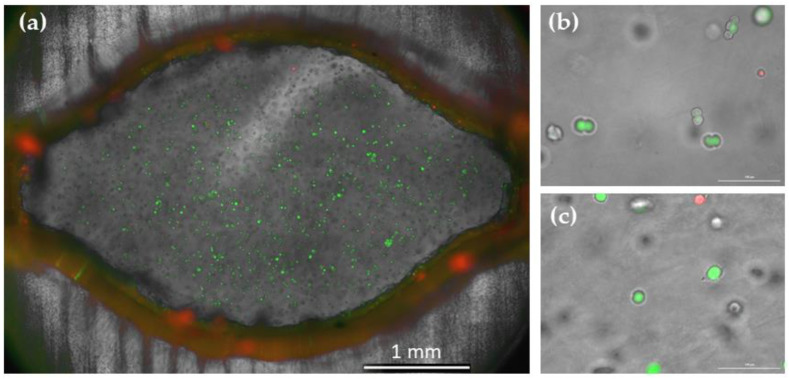
Live/dead staining of hydrogel-embedded L929 cells after three days of cultivation. Overlay of fluorescence (calcein AM/PI) and brightfield microscopic image. (**a**) Image of a whole cultivation chamber; (**b**) Image in a cultivation chamber, scale bar corresponds to 100 µm.; (**c**) Image in 96 well plate, scale bar corresponds to 100 µm. Microscopic images of calcein-AM/PI stained L929 cells on day 3 of the cultivation, bright field image was overlayed with (**a**).

To further investigate a possible influence of the cultivation device on cell viability and as a proof-of-concept experiment for cell staining assays, a live/dead staining of the cultured cells was performed with calcein AM and propidium iodide at the end of cultivation. Representative images of the staining assay results are shown in Figure 4. No difference in viability was inferred from the results of this live/dead staining between the cells cultured in the cell cultivation device and in the well plate; nor did the staining suggest that cell placement within the device impacted cell viability. Therefore, it can be concluded that this cultivation device did not impair cell morphology, growth, or viability—even though it facilitated easy microscopic monitoring of the hydrogel-embedded cells under perfusion, and significantly reduced the required volumes of hydrogel and cell suspension.

## 4. Discussion

The presented 3D-printed microfluidic perfusion system was successfully applied in a proof-of-concept cultivation of hydrogel embedded fibroblasts. The permeable membrane separating the hydrogel compartment from the microfluidic perfusion channel system prevented detachment of the hydrogel, while also facilitating adequate gas exchange and nutrient supply to the cells (as indicated by the microscopic analysis and the cell staining experiments described above). This design also permitted easy microscopic monitoring and staining of the hydrogel-embedded cells, and significantly reduced the required volumes of hydrogel and cell suspension. Since the cultivation device can be easily disassembled, the hydrogels were quickly recovered post-cultivation for further analysis. In principle, this property could even allow researchers to reuse this cultivation device.

To ensure a minimal focal distance in the presented design of the cultivation device, the hydrogel-embedded cells were supplied with perfusion medium from one side only. In the proof-of-concept cultivation of 0.75 million L929 cells per mL embedded in the alginate-based hydrogel presented by Dahlmann et al. [9], no dependency of cell morphology, viability, or growth on the special distance from the perfusion medium chamber was observed. However, the formation of oxygen or nutrient gradients in the hydrogel resulting from the device design could be possible and should be taken into account when using the device. Gradients occur naturally within in vitro tissues of biological organisms, and therefore they may be desirable in some 3D cell culture applications [12,30]. Where such gradients are not desired for an intended application of the cultivation device, however, the course of the perfusion medium channels could instead be rapidly adjusted. The perfusion rate can, of course, also be quickly adapted to suit the purposes and parameters of an intended application.

In order to reduce manual intervention and simplify handling, additional efforts to further automate and miniaturize this system in the future are envisioned. The tubing, medium reservoir, and bubble trap elements could all potentially be fabricated as part of the 3D-printed microfluidic cultivation device—thereby decreasing the required amount of culture medium. This would also potentially mitigate the need for removal of the perfusion system prior to microscopic analysis, thereby also reducing manual efforts and contamination risks at that stage of the process. One proposed approach for integrating a bubble trap into a monolithically 3D-printed cultivation device has already been presented by Beckwith et al. [31].

In many OOC devices presented in the literature, as well as in commercially available systems, tissue barriers are constructed by integrating porous membranes between two compartments of the device [16,32,33,34]. Many simplified models are based on the perfusion of hydrogel-based 3D cultures in cell culture inserts (e.g., Transwell^®^ Inserts). In these systems the exchange of nutrients, metabolites, and gases is not restricted to the medium flow, which is in contrast to physiological conditions in tissues. Furthermore, adhesion forces on the wall of the cell culture insert result in an undefined shape and thickness of the culture. In combination with cell-induced deformation of the hydrogel, this can lead to detachment of the culture from the membrane of the cell culture insert. On the other hand, recovery of cells from completely closed systems, such as those based on multiple channels for integration of hydrogel and culture medium, is often very challenging without destruction of the hydrogel [34]. The cultivation device presented here overcomes these limitations due to the design decisions described above. Another key advantage of the presented device over commercially available chip–based 3D cell culture systems is that its production process allows for rapid prototyping of customized versions of the device. For instance, the additive manufacturing process used to fabricate this device allows rapid modification of the course of the media channels, the hydrogel volume, and the number of enclosed hydrogel chambers. In addition, the integration of sensors, e.g., for the detection of cell metabolites, as previously demonstrated by Siller et al. [35] and Arshavsky-Graham et al. [36], is also feasible. Last but not least, the device presents a promising starting point for the realization of complex fluid flow patterns by integrating valve systems into the setup for automated addition of media supplements, for example, based on the work of Winkler et al. [37].

## 5. Conclusions

The additive manufacturing process used to create this microfluidic cultivation device makes it highly customizable for various applications. It can easily be scaled up to a multi-chamber or parallel perfusion device for screening purposes, and even allows for co-cultures of different 3D cell cultures within a single perfusion system. Additionally, the integration of sensors for the detection of cell metabolites is also feasible. Overall, this system presents a promising starting point for researchers exploring how personalized in vitro cell culture platforms can meet the growing demands of 3D cell culture applications and move ever closer towards the development of in vivo-like organ-on-a-chip systems.

## Data Availability

The data that supports the findings of this study is available from the corresponding author upon reasonable request.

## Supplementary Materials

The following supporting information can be downloaded at: https://www.mdpi.com/article/10.3390/cells12141816/s1, Figure S1: Technical drawing of the top part of the cultivation device; Figure S2: Technical drawing of the bottom part of the cultivation device; Figure S3: Technical drawing of the perfusion adapter; Figure S4: Microscopic image of a whole cultivation chamber; Video S5: Assembly of the cultivation device at the beginning of a perfusion experiment and handling of its adapter in well plate format Video S6: Z-stack of microscopic images of a cultivation chamber; title.
