# Supplementary material for: 3D-Printed Microfluidic Perfusion System for Parallel Monitoring of Hydrogel-Embedded Cell Cultures
Source: Cells. 2023 Jul 9;12(14):1816. doi: 10.3390/cells12141816 (PMC10378615; doi:10.3390/cells12141816)
Supplement: Supplementary file 1 [file cells-12-01816-s001.zip › Supporting information.pdf]

Article

3D-printed microfluidic perfusion system for parallel monitoring of hydrogel-embedded cell cultures

Katharina V. Meyer[1], Steffen Winkler[2], Pascal Lienig[3], Gerald Dräger[3] and Janina Bahnemann[2,4*]

1 Institute of Technical Chemistry, Leibniz University Hannover, Hannover, Germany
2 Institute of Physics, University of Augsburg, Augsburg, Germany
3 Institute of Organic Chemistry, Leibniz University Hannover, Hannover, Germany
4 Centre for Advanced Analytics and Predictive Sciences (CAAPS), University of Augsburg, Germany

* Correspondence: janina.bahnemann@uni-a.de

Supporting Information

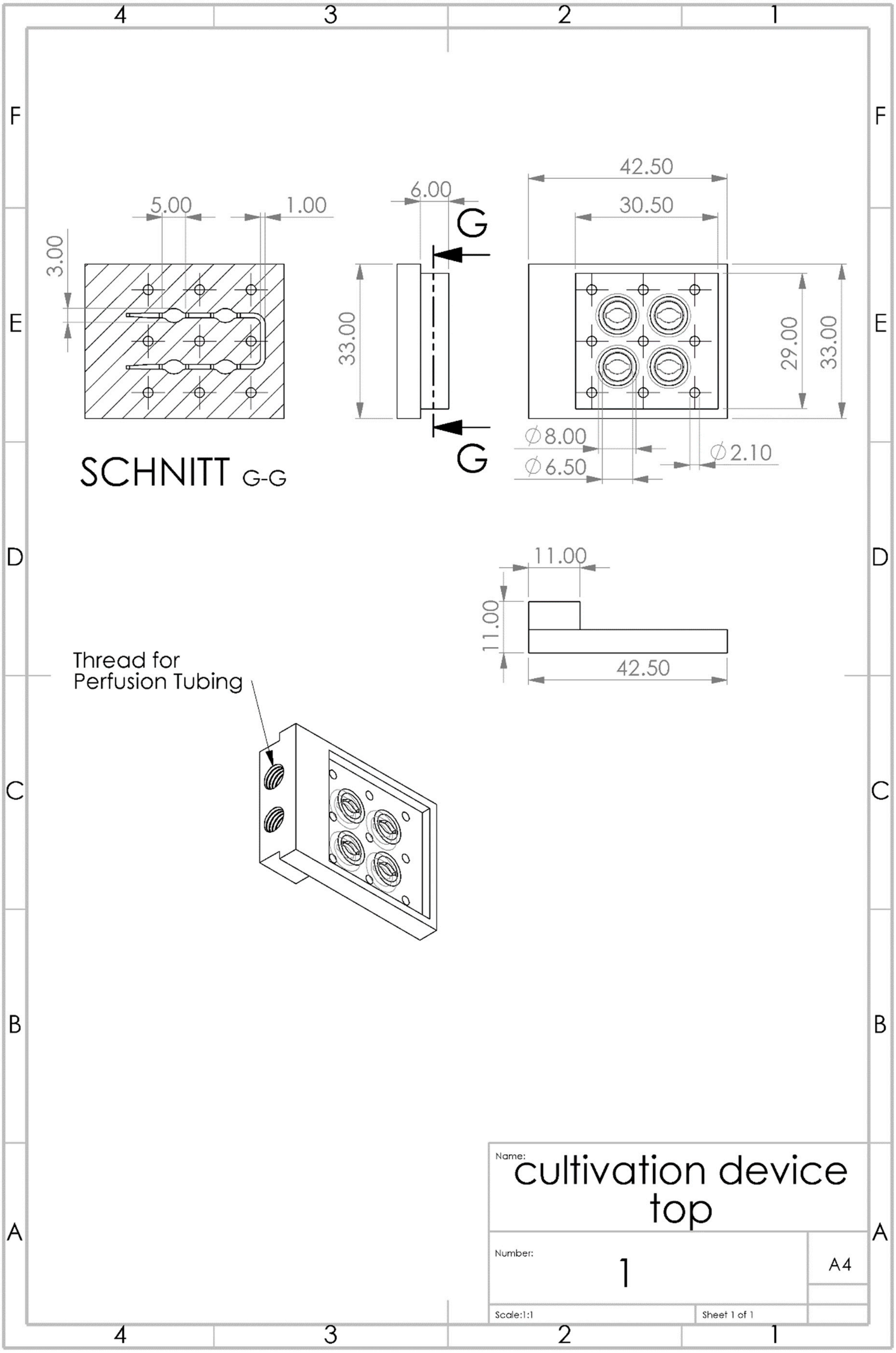

Figure S1: Technical drawing of the top part of the cultivation device.

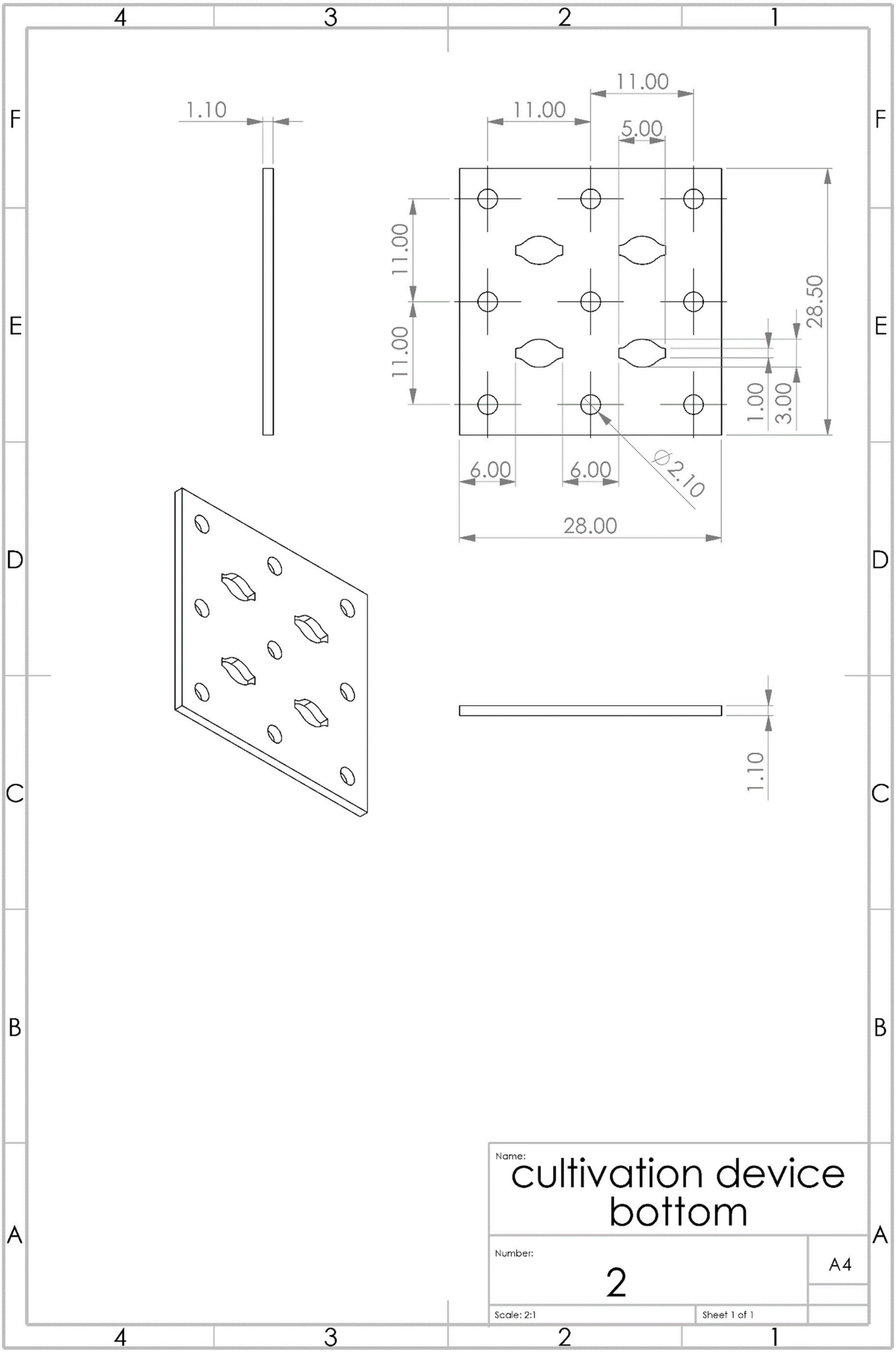

Figure S2: Technical drawing of the bottom part of the cultivation device.

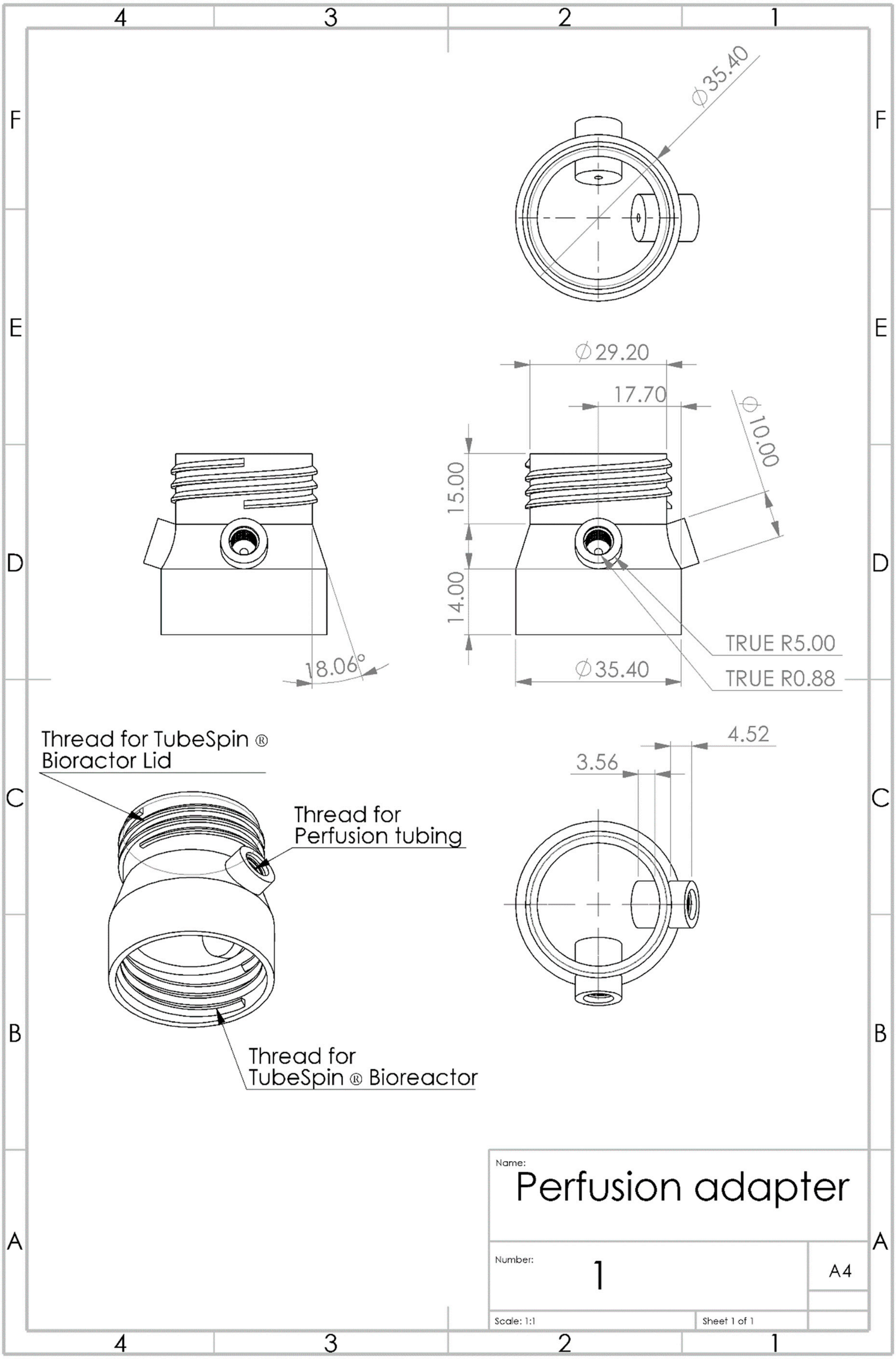

Figure S3: Technical drawing of the perfusion adapter.

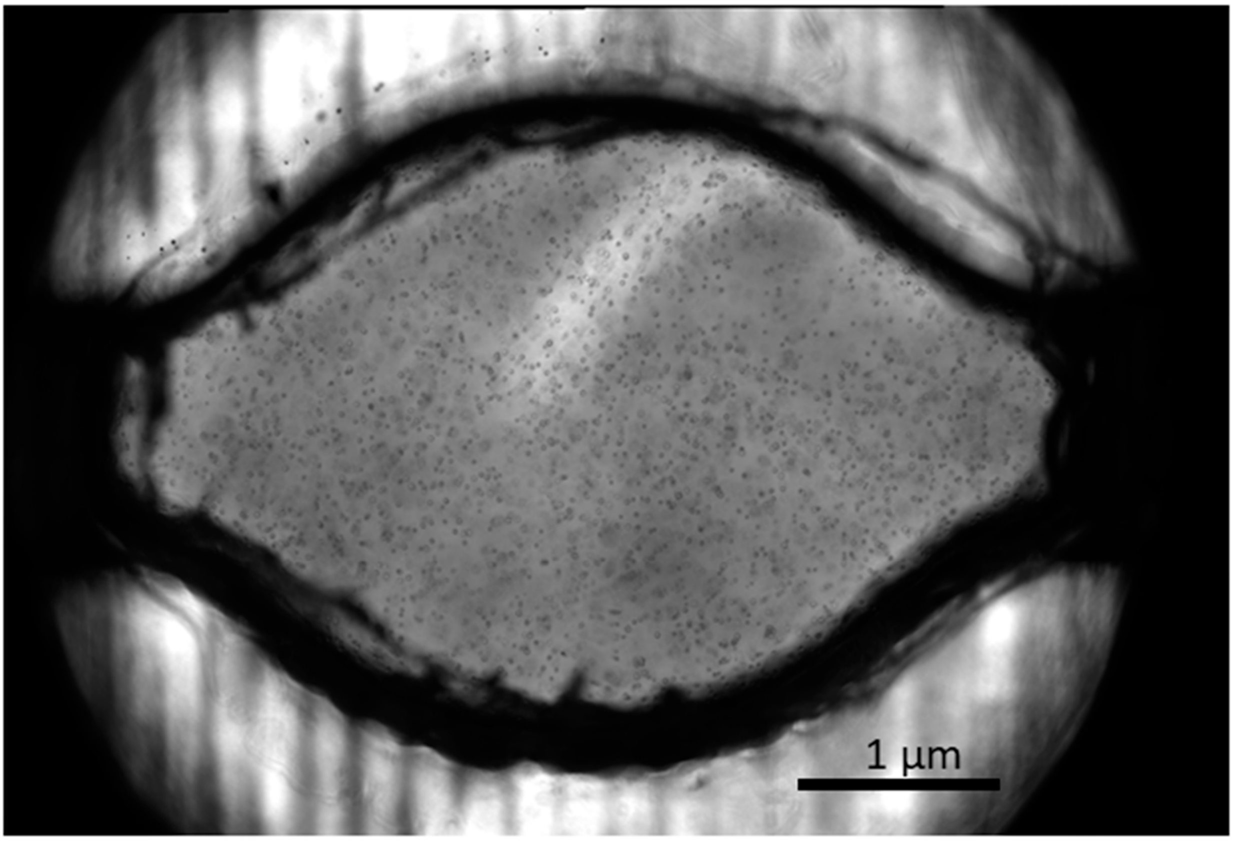

Figure S4: Representative brightfield image of hydrogel-embedded L929 cells in a cultivation chamber of the 3D-printed cell cultivation device. Imaging was performed using a Cytation 5 Cell Imaging Multi-Mode Reader (BioTek Instruments GmbH, Bad Friedrichshall, Germany) at 37 °C. For image stitching the intrinsic function of the Gen5 imaging software was used. The growth behavior of the individual cells did not appear to be dependent upon their position within the cultivation chamber.
